# A mixed methods contribution to the study of health public policies: complementarities and difficulties

**DOI:** 10.1186/1472-6963-15-S3-S7

**Published:** 2015-11-06

**Authors:** Valéry Ridde, Jean-Pierre Olivier de Sardan

**Affiliations:** 1 Department of Social and Preventive Medicine, School of Public Health, University of Montreal, Montreal, Québec, Canada; 2 University of Montreal Public Health Research Institute (IRSPUM), Montreal, Québec, Canada; 3 Laboratoire d'Etudes et de Recherche sur les Dynamiques Sociales et le Développement Local (LASDEL), Niger; 4 École des hautes études en sciences sociales (EHESS), Paris, France

## Abstract

The use of mixed methods (combining quantitative and qualitative data) is developing in a variety of forms, especially in the health field. Our own research has adopted this perspective from the outset. We have sought all along to innovate in various ways and especially to develop an equal partnership, in the sense of not allowing any single approach to dominate. After briefly describing mixed methods, in this article we explain and illustrate how we have exploited both qualitative and quantitative methods to answer our research questions, ending with a reflective analysis of our experiment.

## Introduction

In the mid-nineteenth century, when he became a professor and Dean of the Faculty of Sciences at the University of Lille, Louis Pasteur unambiguously declared his opposition to all scientific dogmatism, despite being one of the great practitioners of experimental science [1]. Since then, the great divide and "epistemological divisions" [2] have long been called into question in scientific circles. In the wars between paradigms, two conflicting methodologies are increasingly rejected: quantitative scientism, which sees only figures and statistical significance as true science; and post-modern relativism, which gives pride of place to narrative. In public health, it is primarily the former that holds sway, under the influence of epidemiological and medical culture. However, it is steadily losing ground. Even the defenders of randomized controlled trials sometimes resort to a qualitative approach, although not always to good effect [3]. Obviously, a few diehards remain who think that data from anthropological interviews are not persuasive enough [4], that "qualitative methods are sort of the least rigorous" [5] or, conversely, that "questionnaire sociology" is "un-questioning", to borrow terms once used by Edgar Morin [6]. A lot of water has flowed under the bridge since researchers started choosing among different methods to answer the questions that have given rise to their research. We are now in an era that is predominantly and rightly characterized by "methodological eclecticism" [7].

In fact, when Abbas Tashakkori and Charles Teddlie [8] published the first edition of their textbook on the use of mixed methods in social science research, they used the image of two bridges--an old one made of stone and a new, metal one--to illustrate the fact that combining quantitative and qualitative methods in the same piece of research is not new. What is new, however, is the attempt to conceptualize it, to propose typologies of how it is exploited, to reflect on the criteria for its scientific legitimacy, and to produce research to that effect. In fact, the study of the use of mixed methods has become a specific field that for several years now has had its own scientific journals (Journal of Mixed Methods Research; International Journal of Multiple Research Approaches) and, since 2013, its academic association, the Mixed Methods International Research Association (http://mmira.wildapricot.org), whose inaugural conference was in 2014.

Hence, recent years have seen a significant growth of interest in the use of mixed methods. This paper will not revisit the conceptual, heuristic and epistemological issues relating to these approaches, since a number of publications have existed for some time that address them in full, both in English [8-10] and in French [11,12]. Suffice it to say that our own approach has many affinities with what has been called "critical realism" [13] or "realist constructivism" [7], a position that refutes positivism as well as post-modernism. Of course, positivism is still powerful, in particular in medical sciences (although one may encounter positivist anthropologists), and post-modernism has developed mostly among social sciences and hermeneutic disciplines (although it has gained some "hard science" supporters). In our view, all social sciences share a specific epistemological background, distinctive from experimental sciences [14]: they are, as Max Weber put it, historical sciences [15]; they are driven by a quest for plausibility (as distinct from falsifiability à la Popper); they construct and formulate, through natural languages, their interpretations of a given reference reality, producing "grounded theory" [16]; and they rely on various methodological procedures for their empirical adequacy. In this view, quantitative and qualitative methods in social sciences are no more than two distinctive specialized sets of procedures, the former being more deductive (based on questionnaires, which imply hypotheses) and the latter more inductive (based on fieldwork, with new research questions continually arising from the field). They mobilize different competencies. Among our team, some of us are quantitativist (public health scholars) and some qualitativist (anthropologists), but we all share the same epistemology, and we have never disagreed on epistemological issues.

Our purpose in this paper is much more pragmatic. It is to show how, in the context of the research programme that has given rise to this supplement, we implemented a mixed methods approach to analyse the public policies of healthcare fee exemptions in Burkina Faso, Mali and Niger, and what we have learnt from it.

Three points should be made, right from the start. First, our research approach was interdisciplinary, in that the mixed methods approach also brought different disciplines, in this case public health and anthropology, into play. Second, our approach was egalitarian, in that it did not give precedence to one discipline or one method over another--something rarely seen in collaborative projects between anthropology and public health [17]. The research problem and research questions (original design of the research), in particular, were framed jointly. Lastly, we developed as pragmatic an approach as possible, in that we were guided in our choice of methods by our questions--and not the reverse, as often happens--and favoured flexibility and adaptation as the research progressed, in contrast to traditional, fixed approaches (to borrow Robson's dichotomy [18]). Furthermore, using mixed methods to study a public policy subject like healthcare fee exemptions in Africa is relatively new. In fact, to the best of our knowledge, only two of the 19 articles published in the period preceding our programme (1988-2009) on this subject adopted such an approach [19].

## Our use of mixed methods

We use Pluye's [11] typology to illustrate our experiment, although it should be noted that researchers in this area have proposed a number of other classifications [8,10,20]. Pluye [11] proposes three types of quantitative-qualitative combinations. The first is the "explanatory sequential", in which quantitative data are collected first, and then qualitative data are used to explain certain quantitative results. In the second, the "exploratory sequential", qualitative data precede quantitative data and orient the production of questionnaires. The terms "explanatory" and "exploratory" borrowed from Pluye [11] are open to question, in the sense that, depending on the particular cases and contexts, both qualitative and quantitative data can either explain or explore. On the use of qualitative approaches for causal analyses, see [21]. Finally, the third type is no longer sequential but "convergent", as the qualitative and quantitative data are produced concurrently, with the results being compared and integrated mostly after the data collection stage, at the time of the analysis.

### Explanatory sequential approach

The first of our examples concerns Burkina Faso, where the research team aimed to measure women's expenses related to childbirth. Since 2006, it has been national policy to subsidize the cost of childbirth in health centres up to 80%, and women are asked to pay a 20% all-inclusive fee of XOF 900 (USD 1.8) for the entire service and accompanying products. Poor women (20%) are normally totally exempted from this XOF 900 payment. Health centres are reimbursed by the State for these deliveries. The research team decided to conduct a quantitative survey of 1,000 parturients to find out from the women themselves what they had really paid. The methodological details and results have been presented elsewhere [22]. The survey involved all women who had given birth in any health centre in Ouargaye district. It emerged that women everywhere claimed to be paying more than they should under existing policy. On average, they said they paid twice the official fixed sum of XOF 900; however, significant divergences were recorded among different health centres. In some cases, the amount was only slightly above the norm, while in others the difference was considerable. Thus, these quantitative data highlighted one gap between policy and actual practice, an "implementation gap" [23], but did not explain the reasons underlying it. Therefore we decided to collect qualitative data to understand these variations. We began by organizing a workshop to share the results with heads of maternity units in all the district's health centres and with members of the district team. The health workers provided some very interesting clues toward a possible explanation. At the same time, however, these workers challenged our findings, as the data highlighted a problem connected with their day-to-day work practices, and they felt challenged by the research results. This collective reaction, in a context in which there is a strong element of social control when group data collection techniques are used, was clearly expected by the researchers [7] and was not new in Burkina Faso [24]. We then conducted 17 in-depth individual interviews with a sample of maternity unit managers, directly on site at their health centres. Not having the means to visit all of the maternity units, we conducted interviews in four of the 25--where the gap between what the women declared they had paid and the official fee was widest--using the extreme case studies method [25,26]. In face-to-face interviews, the health workers soon began to talk more freely, and plausible explanations for the divergences were put forward.

A second example of an explanatory sequential approach in our research programme again concerned Burkina Faso's national policy of subsidising childbirth. The methodological details and results have been presented elsewhere [27]. To measure the effects of the subsidy on health centre use, we studied changes in the average number of deliveries in each maternity unit by means of relatively long time series (six years). Although the introduction of subsidies led to a significant rise in demand, the increase was not the same for all maternity units in the district (the units received no international aid for implementing this policy). Visual and statistical analyses revealed three different scenarios, dividing the units into three categories: a rise in the number of deliveries immediately after the policy's introduction, a rise occurring a few months after implementation and, finally, no change. This analysis was interesting in itself since it showed that it is not sufficient to focus exclusively on the average effects of a policy, but that it is equally important to account for a range of different possible consequences [28]. To understand these differences, we conducted six in-depth case studies. Two health centres were selected for each category. The researcher (Loubna Belaid [27]) then produced a range of qualitative data in each centre (observations, interviews, etc.), remaining on site for approximately two weeks to learn stakeholders' opinions on the observed effects of the subsidy (health workers, women and men from the villages, traditional and religious leaders, etc.). Graphs showing these effects, or their absence, were repeatedly used on site to help interviewees understand the situation. Finally, once these data had been collected, a workshop was organized with all the managers of district health centres to disseminate preliminary findings. This was a way of both sharing results and collecting additional data in the form of stakeholders' reactions. What emerged from this study was that health centres where the policy's effects were immediate enjoyed a combination of favourable factors: strong leadership among health staff, a positive perception by the community of the quality of the healthcare provided, a campaign to present childbirth as being assisted by qualified staff, the appointment of female health personnel, and a relationship of trust between health workers and the public. As for the health centres in which subsidies had a delayed effect, the qualitative study revealed that the effects only materialised after belated changes in health staff and the imposition of a village tax to counter home births. Finally, the subsidies' lack of any impact in some centres was explained by the failure to promote childbirth in health centres, a negative perception by the public of the quality of healthcare, and poor relationships between health workers and the community. This study showed that, although in many cases subsidising costs is a useful strategy for improving access to health services, it is clearly not sufficient on its own, and myriad other factors must be taken into account when formulating a public policy of this kind.

### Exploratory sequential approach

Qualitative data were sometimes marshalled prior to the collection of quantitative data. One example of this was in Niger, where, as soon as we started working on site, all the actors we met complained overwhelmingly of substantial delays in reimbursement for free care administered to children by health centres (Diarra & Ousseini, this issue). We were faced with a real "epidemic" across all the districts and regions where our work took us. Managers decried the serious impact of these delays on the health centres' cash flow. From years of charging for healthcare, health centres and community leaders had amassed substantial amounts of money--although precisely how much remains unknown--and these savings were running out fast as they were used to pay for medicines when the reimbursements failed to arrive. Qualitative investigations undertaken in Mali revealed similar complaints, although the policies there were different. In the context of fee exemptions for treating children against malaria [29], the State in Mali did not reimburse the cost of treatment and inputs but, instead, supplied health centres with medicines that were to be freely dispensed to the patients. Previously, community health centres had bought these same medicines and sold them at a profit. According to local actors, the fact that they were now being dispensed free of charge was having a negative impact on community health centres' financial resources, and State policy-making was damaging the financial health of the Community Health Associations (ASACO), which operate the primary health centres in Mali. To examine more closely the picture painted by the actors and produced with qualitative methods (interviews), we undertook quantitative studies in both Niger and Mali using a model we had tested in Burkina Faso in an earlier research project [30]. We performed accounting analyses in a significant number of health centres in both countries, to the extent possible given the difficulty of obtaining financial documents and the poor quality of record-keeping. The quantitative data produced (e.g. expenses, incomes, profits, cost recovery rate, staff incentives) enabled us to confirm the claims made by actors in Niger and refute those made in Mali. In Niger, delays in reimbursing costs almost spelt disaster for community finances. At the end of 2010, bankruptcy was imminent, and the quantitative study demonstrated that health centres had drawn on their reserves to the point of exhaustion, to cope with government failings. In Mali, on the other hand, the data revealed that complaints made by community health centres leaders were not borne out by the facts, and that community health centres' finances did not suffer as a result of the loss of profits from the sale of anti-malarial medicines because an increase in the number of consultations (for which there was still a fee) made up for the deficit.

### Convergent approach

Finally, the last type of mixed methods used for this programme was the simultaneous collection of qualitative and quantitative data. The methodological details and results have been presented elsewhere [31]. The most interesting example was the study of the workloads of health personnel. In all three countries, exemptions led to increased numbers of visits to health centres and, consequently, to increased workloads. What our research sought to answer was how this increase was perceived by staff and whether it exceeded official labour norms. Thus we gathered both qualitative data, to determine health workers' views on the situation, and quantitative data, to evaluate the true number of hours worked. We also wanted to compare situations in which user fee exemptions were organized with the support of an international NGO (which would guarantee a certain minimum level of service for patients) against situations in which health centres did not benefit from external support of this kind (Olivier de Sardan et al., this issue). For the former, we chose health centres in Burkina Faso in several districts that had been experimenting with fee exemptions for pregnant women and children under five since 2008 and had received generous support from international NGOs, and, for the latter, health centres in Niger, where fee exemptions are institutionalised throughout the nation's health service [32,33]. We set up a specific methodology that involved having observers on site for an entire week in each of the eight centres comprising our sample for each country (four with and four without NGO support) to time and then evaluate staff workload. At the same time, in-depth interviews were conducted with all health professionals. In every case, health personnel complained of an excessive increase in their workload. However, in Burkina Faso the quantitative data contradicted workers' statements, whereas in Niger the situation was ambiguous because of severe staff shortages and shortcomings in the Health Ministry's human resources policy.

## A reflective analysis of the use of mixed methods in our research

The originality of our research methodology undoubtedly lay in the decision to practise all three types of mixed methods research approaches and to adapt them continuously to constraints on the ground, rather than deciding on a particular approach in advance [34]. Pragmatism was at the core of our collaboration.

To start with, we conducted convergent research. Then, as the investigations progressed, we developed a quantitative research framework to answer the questions raised by the qualitative results, and vice versa. However, perhaps our main achievement is that we succeeded in building an equal partnership among researchers coming from the two methodological schools, without either predominating--something rarely achieved in the vast majority of research projects combining quantitative and qualitative methods [35]. In this equal partnership, the team as a whole formulated the starting issue before the investigation stage and then continued to discuss matters together throughout the investigations. In other words, the entire research process (issue, framework and methodology) was developed jointly, from start to finish.

To our knowledge, this type of pragmatism-based experience of interdisciplinary collaboration is relatively rare, as neither the context nor the research culture around health systems and policies in Africa lend themselves to it [36,37]. In Africa, research activities are often not valued [38], and in francophone Africa, in particular, research is often stifled by the hegemony of consultancy activities, which are much more lucrative. The workings of consultancy are very different from those of research and leave little room for innovation and methodological reflection [39]. Research laboratories are rare, and working in teams is seldom done [40]. In interdisciplinary studies in the health field, the health sciences very often play a predominant role, using a few experts in social sciences in a very instrumental and perfunctory way [41,42]. However, in francophone Africa, health anthropology has developed significantly and independently over the past 30 years, paying particular attention in the past 15 years to health policy [43]. Conversely, in Anglophone Africa, medical anthropology remains a weaker discipline, more subjugated to the health sciences, and still largely focused only on popular representations and practices; however, qualitative methods are progressing in the realm of public health, in particular through studies on health systems and policies [44]. In our research program, the health anthropology team at LASDEL offered the advantage of having already worked on the implementation of public policies (not a typical subject of mainstream anthropology). For their part, the public health team from Montreal and their collaborators were also very interested in implementation studies and had already carried out studies along those lines; even there, the approach is not often adopted for implementation studies in public health, where studies on outputs and outcomes predominate. This shared perspective (the analysis of expected and unexpected effects in the implementation of a public health policy) provided us with a solid common foundation for our research. In this respect, it may be that the advancement of mixed methods will not depend primarily on sharing different "toolboxes", but rather will require especially a capacity to unify research questions within a shared issue (in this case, the focus on implementation, the selection of a pragmatic research process, and the same epistemological orientation). The key results, showing that there were simultaneous increases in use and decreases in quality, demonstrate the potency of this collaboration.

Nevertheless, our experiment suffered from certain limitations. The first was deliberate; we opted for a collaboration between two specific networks, one around a Montreal public health team with solid partnership experience in the Sahel region, and the other around a Niamey health anthropology team with many contacts in neighbouring countries. Theoretically, it would have been entirely conceivable to widen our multidisciplinary research program to include other specialties, such as health geography or health economics, in particular. However, these disciplines are not very structured in the three countries being considered, and in conducting this study we wanted to rely essentially on strong local capabilities. Every research project is, likewise, the product of certain choices, to some extent contextual and relational, that exclude other theoretical options (other disciplines, other countries, other definitions of the subject, etc.).

On the other hand, some limitations were internal to the research process and were only revealed through experience. For instance, there was little contact in the field between members of the two groups, and methodological discussions were too restricted, as they were confined to the group leaders. In this regard, it would have been particularly judicious if, at the start of the programme, the quantitative researchers had participated in the anthropologists' preliminary collective investigations [45] and a common methodological workshop had been organized. Also missing was a workshop at the halfway mark to take stock of qualitative-quantitative relations, manage convergences and divergences, and plan the next stages. Cost and logistics were certainly factors in this lack of opportunity for deliberating on methods. Moreover, our approach was complicated by the fact that it covered three countries with different teams, who did not have the full range of expertise: in both Mali and Niger we were unable, or did not know how, to draw on local expertise in quantitative methods. This had to be brought in from Burkina Faso (although local assistants were used) to support the work but also, in a way, to strengthen local capacity.

## Qualitative and quantitative methods in African contexts: bias, advantages and constraints

It is impossible to separate reflections on the methodology of investigation procedures from the matter being investigated [44,46]. Our experiment was specific to the context of research on public policies for health in Africa. Methodological problems must be understood in that context, and, more generally, in the context of public policy-making in Africa. This perspective raises questions about the advantages of the different methodological approaches and the challenges they present.

To produce figures and present graphs is to speak the language of decision-makers and thereby to stand a better chance of being heard by them, given that, in the health field, most of them are physicians who are basically trained in simple quantitative approaches. This is true for any research aimed at improving, for the benefit of users, the way public services work; but because of the importance of this epidemiological culture, it is truer still in the field of public policies relating to health. Figures alone are seen by policy-makers and technical experts as meaningful data--which does not mean, of course, that these officials always take such data into account. Producing a diagnosis of a situation and using that diagnosis to make decisions that can alter that situation are two very different processes. For example, our work revealed that the fee women said they paid to the health centre when they gave birth was well in excess of what was stipulated by the national policy in Burkina Faso [22]. This research confirmed other works, all of which had been made public and communicated to policy-makers. Yet no decisions have been made on the matter. The same applies to the failure to provide free births for poor women [47]. However, the quantitative dimension is not only useful because it is aligned with the quantitative rhetoric of decision-makers, but it is also indispensable in terms of the knowledge it provides.

Nevertheless, in the three countries investigated, producing quantitative data entailed numerous challenges. In the first place, routine data (records, compilations and statistics within the health system) are often difficult to obtain; they are frequently kept by one individual and endless requests for authorization are required, even when ethics committees have given consent. Once obtained, they take a very long time to process (in particular, because of large amounts of missing or incorrect data) before they can be used. On top of that, they are very often unreliable, such that any analyses must be interpreted with caution. Sometimes they are also fragmentary, even non-existent, as our investigations into the accounting records of the management committees (COGES) revealed in certain cases [48]. Still, these routine data can be useful, given the time and means to verify their quality [49].

On another front, situations in which questionnaires are administered can confuse or worry respondents and lead to distorted responses, especially if--as is often the case--respondents feel they may be personally affected by the answers they give. In such cases, observational data, not often used, should be encouraged (even if the presence of observers can influence matters) and given precedence over declaration data, which predominate. In both cases, however, producing quantitative data is expensive and requires paid interviewers, whose work must be closely managed and supervised; results are often skewed when interviewers are poorly motivated and sometimes incompetent, negligent or dishonest. The quality of data collected via questionnaires is a major concern that is not always given sufficient attention when those questionnaires are being designed. Analysis of data is complex, technical and difficult to share with qualitative researchers who have not been trained in quantitative methods. Researchers who keep their skills up to date and are able to perform such analyses are few and far between. Finally, analysing these data often requires considerable time, since before reaching valid conclusions--bearing in mind the data quality challenges mentioned earlier--researchers (if they are thorough) must scrupulously check the results. As such, it is not uncommon for results to remain unavailable until a year or two after the data have been collected (the best case scenario), and thus to be presented long after they would have had the potential to influence policy decisions.

In the domain of public policy-making, the difficulties of implementing policies are grossly under-estimated, played down or glossed over. The "implementation gap" receives scant attention in Africa [50]. The official channels for gathering and reporting information fall foul of conflicts of interest: it is obviously not in a public official's interest to highlight shortcomings at either his level or the lower levels under his supervision. Speeches, figures and official reports, therefore, tend to focus on activities carried out and targets achieved. Whether in African administrations, international institutions, cooperation agencies or NGOs, complacency usually predominates. Defence of the policy being pursued or the reform under way is the order of the day. Experts and consultants can, of course, highlight certain problems, but time constraints generally do not permit them to probe deeply, to the extent of going into the field and conducting investigations at user level. On top of that, there is strong pressure on consultants to practise self-censorship or to tone down their reports.

To put it another way, there is little room in public discourse for malfunctions, failures and departures from norms. Yet these malfunctions, failures and departures from norms exist. Understanding them is indispensable if there is to be any health policy reform, improvement in healthcare quality or strengthening of the health system. There are two ways of approaching them: through daily work practices (using extended participant observation) or through private conversations (involving dialogue, trust and readiness to confide). These two approaches are precisely the core areas of expertise in the qualitative methods developed by anthropologists and increasingly being used by other social scientists, such as sociologists or political scientists.

During intensive fieldwork, data are generated from private conversations and everyday situations, through know-how, patience and rigour. However, once the research process is finished, social scientists subsequently bring their findings into the public arena through reports and publications, inevitably producing some negative reactions (Olivier de Sardan, this issue).

Qualitative methods are frequently disorientating for public health specialists of the old school as well as for decision-makers accustomed to routine quantitative protocols: they involve neither predetermined sampling nor statistical representativeness. Instead, the talk is of triangulation, saturation, case studies, shared representations, organizational cultures, practical norms and relevant statements. Although scientific rigour is achieved through procedures that are very different from quantitative analyses, this does not make qualitative analyses in any way inferior to their quantitative counterparts [7,51,52]. Qualitative data are just as persuasive as quantitative data, albeit in a different way. These are two different regimes of evidence. However, focusing qualitative investigations on the implementation gap of public policies can obscure the positive cases and thereby create a kind of negative bias, as happened, for example, in Burkina Faso [27]. Interestingly, this criticism is often expressed by the very people who would be embarrassed by the exposure of malfunctions. In fact, analyses of malfunctions spark contrasting reactions among public policy actors. Reformers welcome such analyses, which they see as equipping them with the tools for improving or changing public policies, whereas conservatives challenge those analyses or try to discredit them. The search for positive cases should nonetheless feature on the qualitative research agenda, with the understanding that they are more difficult to study (and sometimes even to find) than one might think.

The main challenge posed by qualitative methods lies in the level of the expertise required. Anthropologists generate their own data through repeated interactions on a personal level with the subjects of their investigation. As this is a skill which can only be attained through years of practice, it is difficult to find anthropologists who are highly trained. Yet the quality of the data is dependent to a large extent on the quality of the researcher. This problem is compounded by the growing popularity in Africa of qualitative research "at a discount", which often proves irresistible to technical experts and policy-makers, but does not guarantee the requisite rigour and is unable to precisely document the malfunctions, failures and departures from norms to which we referred earlier. It usually is limited to a few interviews with resource persons or involves resorting to quick standard investigations ("rapid" or "participatory-rural appraisals", or "household economy appraisals", among many others) or extensive use of focus groups.

## Conclusion

Public policies, whether in health or other domains, are unquestionably fertile ground for mixed methods. Yet the use of mixed methods for the analysis of public policies in Africa (and especially French-speaking Africa) remains the exception, as is true elsewhere in the world, as well. For example, in a random sample of articles published in the major international science journals for the study of public policy (sociology and political science) between 2001 and 2010, 72% used quantitative methods and only 1% mixed methods [53]. A concerted effort should certainly be made without further delay by universities to provide in-depth training and by publishers to produce textbooks to support the teaching of mixed methods [11,20,54]. Moreover, the teams who take on the challenge of bringing together quantitative and qualitative researchers rarely go through the reflective process of analysing their experience and sharing the lessons learnt. Rawat et al. [55] offer a very interesting example of such analyses on questions of method. Mixed methods encounter many challenges, of which, in our view, the most interesting relate to quality. There is no question of combining low quality quantitative methods with low quality qualitative methods. Public policies deserve the best on both sides.

## Competing interests

None

## Authors' contributions

VR and JPOS conceived the idea, wrote the draft and final version of the manuscript. All authors read and approved the final manuscript.

## Declarations

This article has been modified from the chapter **Conclusion: Une contribution des méthodes mixtes à l'étude des politiques publiques: complémentarités et difficultés** by V. Ridde and J.-P. Olivier de Sardan in the book *Une politique publique de santé et ses contradictions. La gratuité des soins au Burkina Faso, au Mali et au Niger*, J.-P. Olivier de Sardan and V. Ridde (eds), 2014, Karthala, Paris [56], with the permission of the publisher. The publication of this supplement was funded by a grant from International Development Research Centre (IDRC), Ottawa, Canada.

This article has been published as part of *BMC Health Services *Research Volume 15 Supplement 3, 2015: User Fee Exemption Policies. The full contents of the supplement are available online at http://www.biomedcentral.com/bmchealthservres/supplements/15/S3.
